# Supplementary material for: Loss of neuronal population organization links pathology to behavior in a model of Alzheimer’s disease
Source: bioRxiv. 2026 Mar 18:2026.03.18.712735. Preprint. [Version 1] doi: 10.64898/2026.03.18.712735 (PMC13015478; doi:10.64898/2026.03.18.712735)
Supplement: Supplement 1 [file NIHPP2026.03.18.712735v1-supplement-1.pdf]

bioRxiv preprint doi: https://doi.org/10.64898/2026.03.18.712735; this version posted March 18, 2026. The copyright holder for this preprint (which was not certified by peer review) is the author/funder, who has granted bioRxiv a license to display the preprint in perpetuity. It is made available under aCC-BY 4.0 International license.

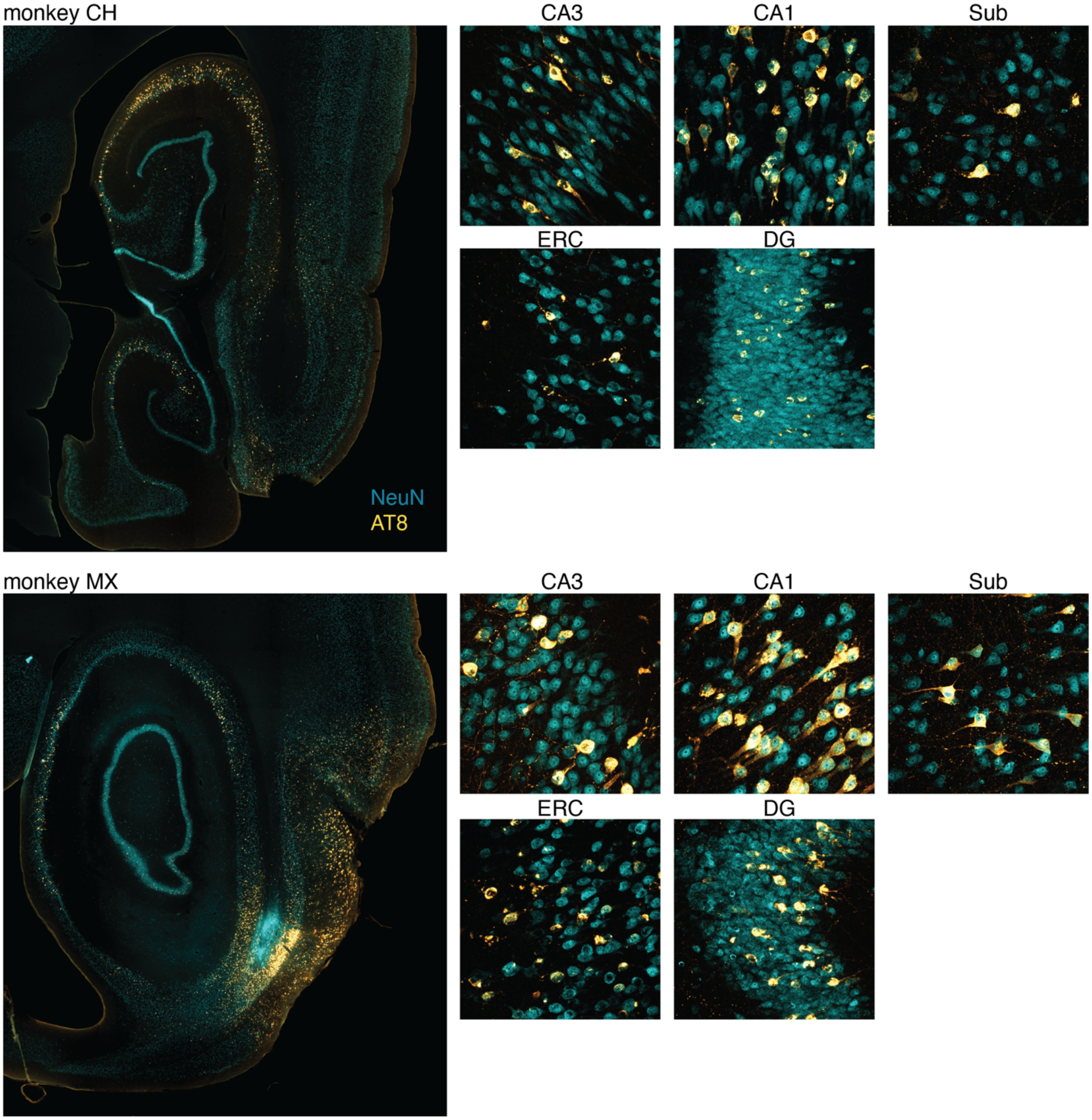

Supplementary Figure 1. AAV-2xTau injection in the entorhinal cortex (ERC) induces robust tau propagation. Histology performed one year after viral injection of dual tau mutation in the ERC. AT8 (gold) was used as a marker for pathological tau spread, with NeuN (blue) labelling neuronal nuclei. No AT8 labelling would be expected in these animals in the absence of the AAV-2xTau injection (see (Beckman *et al.*, 2021; Beckman *et al.*, 2024)). Left hemisphere section shown for each animal. AAV injections were bilateral and tau was confirmed in both hemispheres. Increased magnification portions of the larger (left) image are shown for hippocampus (CA1 and CA3), subiculum (Sub), entorhinal cortex (ERC), and dentate gyrus (DG).

bioRxiv preprint doi: https://doi.org/10.64898/2026.03.18.712735; this version posted March 18, 2026. The copyright holder for this preprint (which was not certified by peer review) is the author/funder, who has granted bioRxiv a license to display the preprint in perpetuity. It is made available under aCC-BY 4.0 International license.

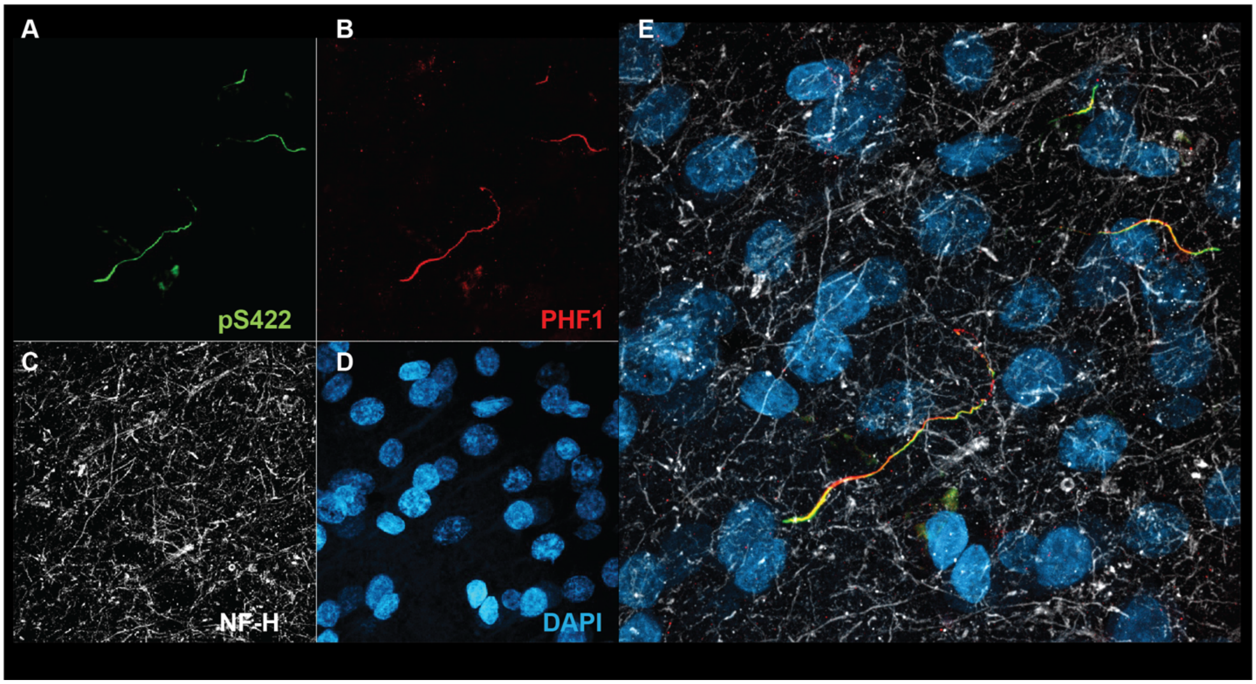

Supplementary Figure 2. Elaboration of histological observations in V4. The same field of view from Figure 1C is shown by compartment for tau markers A) pS422 and B) PHF1, C) Neurofilament Heavy, NF-H, which labels neuronal cytoskeletal elements, particularly in axons, and D) DAPI, which labels nuclei. E) shows panels A-D combined.
